# A Case of Splenectomy, with Remarks

**Published:** 1901-08

**Authors:** J. P. Bryson

**Affiliations:** St. Louie, Mo.


ATLANTA

### Journal-Record of Medicine.

Successor to Atlanta Medical and Surgical Journal, Established 1855, and Southern Medical Record, Established 1870.

VOL. III. AUGUST, 1901. NO. 5.

BERNARD WOLFF, M.D., EDITOR, Nos. 319–20 Prudential.

Published Monthly.

M. B. HUTCHINS, M.D., BUSINESS MANAGER, No. 64 Marietta St.

### *ORIGINAL COMMUNICATIONS.*

### A CASE OF SPLENECTOMY, WITH REMARKS.*

BY J. P. BRYSON, M.D.,
ST. LOUIS, MO.

Splenectomy is still a sufficiently rare operation to justify the report of a single case, all the more so when the patient is the subject of leucemia, a condition distinctly contraindicating surgical intervention, except for the most urgent reasons.

The patient, Jane McK., aged twenty-seven years, American, single, without occupation on account of her disease, residing in Vienna, Mo., was seen September 25, 1900, on account of what was supposed to have been an enlarged and floating kidney. Physical examination quite easily demonstrated that the large tumor occupying the left side of the abdomen and extending from above the lower border of the rib to and into the pelvis, was not a kidney at all, but a very much enlarged and freely movable spleen. I quote now from the hospital records:

* Read before the Medical Society of City Hospital Alumni, February 21, 1901.

*Family History.*—Father living and healthy at the age of seventy years, mother sixty-one years of age. Five brothers and one sister.

*Past History.*—Usual diseases of childhood; mumps at eight years, pneumonia at fourteen years. Menstruation began at seventeen years; has always had dysmenorrhea. Her present trouble began in early girlhood with pains of a dull, heavy character in the left side, most severe in the left inguinal region. For a long time she had periods of perfect comfort, but about one year ago pains become more severe, recurred more frequently, lasting two or three days, with intermissions of the same duration; she had occasional hot flushes, vertigo and persistent cephalalgia.

For the last three years her feet and legs, as far as the knees, became much swollen, blue, and even black, painful to the touch, pitting on pressure; no varicose veins.

Examination showed a large tumor on the left side, extending from beneath the ribs down into the bony pelvis. The tumor is quite movable, changing position with the patient's movements, not painful on manipulation, quite firm, round and smooth. No glandular enlargement and no enlargement of the superficial veins.

*Urinalysis.*—S. G. 1020, dark amber, clear, acid, no albumin, no sugar, no bile, no excess of indican; microscopically negative.

October 12. Operation. An incision eleven and a half inches in length was made from the free margin of the ribs in a straight line parallel with and two inches to the left of the median line, exposing a spleen enormously enlarged, capsule smooth and mottled, showing the thickening of an old perisplenitis, quite dark and congested.

The pedicle was long enough to permit of delivery of the organ. The splenic artery was large, the vein greatly distended, admitting the tips of three fingers on section. The pedicle was tied off in sections; twelve ligatures of No. 9 braided silk being used, the distal end was clamped and the organ removed. At the time of the removal the organ weighed five pounds and two ounces. The wound was closed and the patient put to bed; she rallied well; saline enemata administered; no shock.

October 13. Comfortable, except for gaseous distention; sleeps well.

October 17. Superficial sutures removed, wound healing; blood examination for plasmodia negative; leucocytes and eosinophiles increased; yeast fungi and mould present.

October 18. Epistaxis and bleeding from the gums. Anterior nares plugged.

October 20. All sutures removed.

October 24. Temperature has gradually risen to 103° F. Diarrhea. Blood again examined—no plasmodiæ found; leucocytes still increased in proportion to red cells. Urine for twenty-four hours, seventeen and a half ounces.

October 25. Urinalysis: S. G. 1030, faintly acid, 2 per cent. of albumin, no sugar, some epithelial and mucous cells, urates in excess, yeast fungi and moulds and granular casts; few red blood cells.

October 26. Urinalysis: S. G. 1020, acid, dark amber, slightly cloudy, ½ per cent. of albumin, no sugar, mucous and flat epithelial cells, few red blood cells; calcium oxalate crystals, yeast moulds and some granular casts.

October 28. Urinalysis: S. G. 1010, faintly acid, amber, clear, ¾ per cent. of albumin, no sugar, many bacteria, yeast moulds and a few faintly granular casts. Diarrhea continues.

November 1. The patient very comfortable. Diarrhea has ceased.

November 12. Blood examination: Leucocytes still increased, but not as largely as before; eosinophile more abundant, no plasmodia.

November 15. Urinalysis: S. G. 1020, clear, acid, no albumin, no sugar, some mucous cells, calcium oxalate crystals and a few bacteria.

November 19. Discharged from the hospital.

November 23. Blood examined: No plasmodia, very few eosinophiles, white cells not as numerous as before. The patient appears well. No enlargement of the thyroid or lymphatic glands.

### RÉSUMÉ.

A large, well-developed woman, aged twenty-seven years, with good family and personal history, but presenting evidences of congenital floating spleen which had become hypertrophied, gradually developed symptoms of obstruction, anemia, dysmenorrhea and intermittent amenorrhea. Within the past year crises of acute pain and shock, becoming more and more severe and frequent, referable to axial rotation of the enlarged spleen, have developed. Absence of lymphatic involvement. Splenectomy followed by neither shock nor hemorrhage. On the sixth day epistaxis and bleeding from the gums, which was quickly followed by pyrexia, anorexia, diarrhea and acute nephritis, all of which subsided within twelve days. Recovery is complete and the anemia is distinctly improved two months after operation. During convalescence no enlargement of the lymphatic tissues is demonstrable.

As may be seen, there is an unfortunate hiatus in the record, viz., the absence of a competent blood examination before operation. On September 25th, when the urinalysis was made at my office, the blood was, as I recall, also examined, and I got the impression from a verbal report that, while there was a high grade of anemia, there was not a true leucemia. Even in the face of such contraindications there was urgent need for relief, which could only come from operation. The case seemed to be one of congenital wandering spleen, enormously enlarged, and developing symptoms due to displacement, obstruction, pressure, dragging on certain viscera and crises referable to axial rotation. Only after the occurrence of two such crises, causing dangerous symptoms of collapse, was it determined to face the risk of splenectomy.

Both of these crises appeared to be due to rotation of the enlarged organ, the lower border of which slipped over beyond the median line, at the same time that it greatly increased in size and became tender to pressure. Quickly there followed nausea, emesis, a heavy dragging feeling in the epigastrium, rapid pulse, cool clammy skin and great pallor. Under such conditions one may be justified in taking considerable risks, as when splenectomy is done in those cases of acute anemia due to lacerations and internal hemorrhage with collapse. Moreover, the pressure symptoms manifested by edema and hyperesthesia of the lower extremities and symptoms of obstructive constipation were not only increasing, but seemed to account, in a measure at least, for the anemia.

Leaving aside the barbarous practice alluded to by Dionis (1733)

as unmilting, and the operations for disease said to have been done by Zaccarilli in 1549, and by Ferrerius in 1711, both of which have been discredited, it seems that in late years surgeons have done splenectomy for the following conditions: First, leucocythemia; second, injury or prolapse; third, certain cases of movable spleen; fourth, simple hypertrophy with or without cirrhosis; fifth, sarcoma or lymphosarcoma; sixth, cysts; seventh, hydatid disease.

The operation for leucocythemia is now considered unjustifiable because of its high mortality (over 90 per cent).

Writing in 1896, the late Mr. Greig Smith was able to collect the following summary of tables: Collins, 89 cases, 13 for diseases not associated with leucocythemia; of these 8 recovered. So far there appears to have been done only one successful operation for leucocythemic spleen—that of Franzolini of Turin, and this is doubtful.* Ashurst, 43 for disease, with 31 deaths; 21 for injury or prolapse, all successful.† Nussbaum, 26 cases for traumatic causes, with 16 recoveries. Gilson, 18 operations for injury, all recovered; 37 for disease, with 29 deaths and 8 recoveries.‡ Podrez and Kharkoff estimate the total mortality at 73 per cent. Mollieret, 28 cases for disease and 11 for wounds, with same results as above.§ Wright, of Manchester, England, tabulated 62 cases; 22 for leucemia, all fatal; 23 simple hypertrophies, with 15 deaths; 7 for malaria, with 2 deaths; 3 for cystic disease, all recovered.|| Asch, 90 cases, 51 successful (14 for wandering spleen).¶ Dr. M. Howard Fussell, of the University of Pennsylvania, collected statistics up to 1890, showing a total of 105, with 57 recoveries and 48 deaths; 28 were for simple hypertrophy, with 19 deaths; 24 for leucemia, with 23 deaths; 26 for accident, with 1 death; 16 for floating spleen, with 1 death; 5 for cyst of spleen, with 1 death. The rest for rupture, suppuration, pernicious anemia and sarcoma. Spanton has collected 25 cases for leucemia, with 24 deaths; 75 for non-leucocythemic spleen, with 28 deaths.** Dr. Richard Douglas, 194 cases: for

* Med. Woch, No. 20, 1883.

† International Encyclopedia of Surgery, Vol. V.

‡ Rev. de Surg., April 10, 1885.

§ Diction. Enc. des Sc. Med. 1883.

|| Med. Chron., December, 1888.

¶ Inter. Journ. of Med. Sci., November, 1888.

** Brit. Med. Jour., November 2 and 9, 1895.

leucocythemic spleen, 36 splenectomies, with 31 deaths; simple hypertrophy, 59 operations, with 25 deaths; for neoplasm, 5 operations, with 3 deaths; for hydatids, 6 operations, with 2 deaths; for wounds, 43 operations, with 11 deaths.*

Summing up, Greig Smith† says: "Operations for leucocythemic spleen are unjustifiable; operations for traumatic lesions are justifiable and safe. For movable spleen excision ought not to be carried out till less severe measures, such as mechanical support or operative fixation, have been tried and found ineffectual. For cysts, the spleen may be removed with a fair chance of success, but puncture or incision with drainage ought to have a trial first. In the early stage of malignant disease the operation is justifiable. In the rare cases of primary hypertrophy the operation is permissible if the disease is attended with danger or serious discomfort."

In the case of cysts, abscess and certain cases of wandering spleen, the trend of opinion seems to be toward splenectomy rather than splenopexy, or incision and drainage. In this surgical opinion seems to run parallel with that in regard to nephrectomy as related to nephrotomy and nephropexy in certain conditions.

Plucker* estimates the mortality of splenectomy for certain conditions, as follows: Leucemia, over 90 per cent.; essential hypertrophy, 57 per cent.; malarial hypertrophy, 55 per cent.; hydatids, 40 per cent.; sarcoma, 30 per cent.

So far as I have been able to see, the estimates of mortality have been based on the figures given above, but it is encouraging to note that Burtz has demonstrated that the mortality from splenectomy has, in recent years, been diminished, which he thinks may be attributed to improved technique, asepsis and a proper restriction of the cases in which it should de done.

Up to this time only a few splenectomies for floating spleen associated with twisted pedicle causing pain and shock have been recorded. One such case by Dr. Isaac Scott Stone may be found reported in the *Annals of Surgery*, Vol. XXX., page 321, 1899. So far as I have been able to ascertain no splenectomy has been done in such cases associated with splenic leucemia.

---

*Journ. of the Amer. Med. Ass'n, April 25, 1896.
†Abdominal Surgery, Vol. II, page 1093.

The report of the pathologist which follows, demonstrates that the leucemia in the case reported here was not myelogenous or lymphatic, but splenic. While sufficient time has not elapsed to determine the ultimate result, the case may assist in modifying somewhat the sweeping generalization against splenectomy in cases associated with leucocythemia because of the high mortality, as well as in explaining the few successful operations done in such cases, operations which have been discredited, perhaps unjustly.

MICROSCOPICAL REPORT OF DR. T. KODIS.

The microscopic examination of the spleen shows great changes in the structure of the tissue. The follicles are absent and the pulp is replaced by connective tissue and blood-vessels. The section has a great quantity of large vessels with round dilatations on the end. Their wall is very thin and covered by endothelia and in some places by spindle-shaped cells. These round and oval dilatations at the blood-vessels are Thoma's ampullæ. The connective tissue is very much increased over the normal quantity; we see between its fibers a great many round cells with characteristics of large lymphocytes. The eosinophile cells and myelocytes, the same cells we find in capsules of the spleen and in the trabecula.

Such changes in the tissue of the spleen might be present in the following pathological conditions: First, primary megalospleny; second, chronic stagnation of the blood, as in cirrhosis of the liver, and in heart and lung diseases; third, in malaria; fourth, in chronic leucemia. The primary megalospleny shows large cells inside the dilated vessels and in the walls of them. We do not see any such cells in our specimen. The stagnation of the blood and malaria is excluded by the absence of the corresponding clinical symptoms and by lack of deposit of pigment which is always present in old cases of malaria. The infiltration of the connective tissue and of the capsule by lymphocytes shows that we have to do here with an old case of leucemia where the hypertrophic conditions of the adenoid tissue disappear and the connective tissue becomes hypertrophied.

---

NOTE.—The patient presented herself at my office June 7, 1901

* Deutch. Med. Woch., August 12, 1897.

Five weeks before she had been thrown from a horse sustaining a severe contusion of the right shoulder which was also dislocated. Her health had been excellent and she appeared strong and well except for some stiffening and thickening about the shoulder. There was nothing in her general appearance to indicate that she had ever been the subject of leucemia.

At my request Dr. Given Campbell made an examination of the blood. The report follows:

BLOOD EXAMINATION REPORT OF DR. GIVEN CAMPBELL.

The patient was seen June 7, 1901, and her blood examined; result of examination was as follows: Blood normal in color but seems a trifle thin in consistence. At the point of puncture the blood flowed unusually profusely. Hemoglobin estimated, by Hammerschlag's method, reveals 80 per cent., which is very little, if any, lower than is normal for the average American woman. Erythrocytes are normal in size and shape and show no nucleated corpuscles. A study of the white cells shows no increase of either large or small lymphocytes. Polynuclear neutrophiles are normal in number, but the nuclei of these contain some peculiar granules which take the same color as the chromatin but stain more diffusely; they have been present in several specimens of blood, one of which was taken as early as twelve days after operation. The eosinophiles are present in increased numbers, constituting about ten per cent of the total leucocytes; they are all of the polynuclear variety. A careful examination fails to reveal the presence of any myelocytes, nor does the blood now show any signs of leucemia.

